# Racial and Ethnic Disparities in Cesarean Birth Trends in the United States

**DOI:** 10.1001/jamanetworkopen.2025.44078

**Published:** 2025-11-17

**Authors:** Marie J. Boller, Bharti Garg, Hailey A. Baker, Maria I. Rodriguez, Nicole E. Marshall, Aaron B. Caughey

**Affiliations:** 1 Division of Maternal-Fetal Medicine, Department of Obstetrics and Gynecology, Oregon Health & Science University, Portland; 2 Department of Obstetrics and Gynecology, University of Minnesota Medical School, Minneapolis; 3 Division of Complex Family Planning, Department of Obstetrics and Gynecology, Oregon Health & Science University, Portland

## Abstract

**Importance:**

Inequity by race and ethnicity persists in obstetric practice. A comprehensive comparison of the rates of cesarean birth by race and ethnicity in the United States is essential to target quality improvement efforts.

**Objective:**

To evaluate the trends in national cesarean birth rates stratified by race and ethnicity as well as parity from 2012 to 2021 and to describe any racial and ethnic disparities.

**Design, Setting, and Participants:**

This was a retrospective observational cohort study of birth data from the National Vital Statistics System. Inclusion criteria were singleton, nonanomalous, full-term gestation (37 weeks and 0 days to 42 weeks and 0 days) births with vertex presentation. Trends in the proportions of cesarean births from 2012 to 2021 among nulliparous individuals, multiparous individuals without prior a cesarean birth, and multiparous individals with prior a cesarean birth were graphically examined. Data were analyzed from March 27, 2024, to July 13, 2025.

**Exposure:**

Race and ethnicity, self-reported as American Indian or Alaska Native, Asian, Hispanic, non-Hispanic Black, and non-Hispanic White.

**Main Outcomes and Measures:**

Main outcomes were cesarean birth rates among nulliparous individuals and multiparous individuals with or without a prior cesarean birth. Cesarean birth rates among self-reported racial and ethnic groups were compared in 2021 relative to 2012 using risk ratios (RRs). Further, the association of racial and ethnic groups with cesarean deliveries was examined using multivariable Poisson regression analyses adjusting for maternal age, education, insurance, prepregnancy body mass index, diabetes (preexisting and gestational), hypertension (chronic and gestational), birth weight, and gestational age and reported as adjusted RRs (ARRs).

**Results:**

In total, 30 014 020 births were included (mean [SD] maternal age, 28.6 [5.8] years). For non-Hispanic Black individuals, the adjusted risk of cesarean birth was higher compared with individuals from other racial and ethnic groups; this disparity increased from 2012 to 2021 (2012 ARR, 1.12 [95% CI, 1.11-1.13]; 2021 ARR, 1.17 [95% CI, 1.14-1.20]). This finding persisted among nulliparous (2012 ARR, 1.20 [95% CI, 1.17-1.24]; 2021 ARR, 1.23 [95% CI, 1.19-1.27]) and multiparous individuals without a prior cesarean birth (2012 ARR, 1.32 [95% CI, 1.20-1.45]; 2021 ARR, 1.33 [95% CI, 1.24-1.43]).

**Conclusions and Relevance:**

In this cohort study of births in the United States from 2012 to 2021, the rate of overall cesarean births decreased slightly over the study period. However, racial and ethnic disparities persisted, with increasing risk of primary cesarean births among non-Hispanic Black individuals compared with individuals from other racial and ethnic groups. Quality improvement efforts to reduce unnecessary cesarean births should address this inequity and the structural racism that drives it.

## Key Points

**Question:**

In the United States, what are the recent trends and disparities in cesarean birth by race and ethnicity, stratified by parity?

**Findings:**

This cohort study included 30 014 020 births and found that the rates of primary cesarean birth among non-Hispanic Black individuals increased compared with individuals from other racial and ethnic groups in 2012; this disparity widened from 2012 to 2021.

**Meaning:**

These findings suggest that systemic racism in obstetrics continues to shape trends in cesarean births and must be addressed directly by quality improvement efforts to safely prevent unnecessary cesarean births.

## Teaser

This cohort study evaluates trends in the rate of cesarean births by parity, previous cesarean delivery, and race and ethnicity.

## Introduction

Cesarean births, lifesaving when indicated, drive excess morbidity and mortality when performed with undue frequency.^1,2^ In the United States, structural inequity impacts obstetric morbidity and mortality; rates of maternal death are highest among American Indian and Alaska Native individuals and non-Hispanic Black individuals,^3^ and cesarean-related morbidity is observed at higher rates among individuals self-identified as non-Hispanic Black and Hispanic.^4^ The cesarean birth rate in the US increased from 1 in 5 in 1996^5^ to 1 in 3 in 2011. In 2014, the Society for Maternal-Fetal Medicine and the American College of Obstetricians and Gynecologists published an obstetric care consensus document with labor management guidance aimed at safely reducing the rate of primary cesarean births.^6,7^

Efforts to decrease the rate of first cesarean births in the US have focused on nulliparous, term, singleton vertex cesarean births as a quality improvement metric that excludes common indications for cesarean birth and captures a low-risk population.^8,9^ Although the ideal cesarean rate is unclear,^10^ wide variation from 4% to 70% exists between institutions within the US,^11,12,13^ indicating that structural factors may influence the rate of cesarean births.^14,15^ The US Centers for Disease Control and Prevention established a target nulliparous, term singleton vertex cesarean rate of 23.9% as part of their Healthy People 2020 objectives^16^ and a target of 23.6% for Healthy People 2030.^17^

To evaluate cesarean birth rates beyond the nulliparous, term, singleton vertex population, the Robson cesarean classification system offers a comprehensive framework inclusive of birthing women that has been endorsed by the World Health Organization^18^ and used outside the US but not adopted widely in the US.^19^ This system groups births into 10 mutually exclusive categories using 6 obstetric variables: parity, prior cesarean birth, gestational age, multiple gestation, fetal presentation, and labor onset.^20^

An additional key metric in the assessment of cesarean birth rates is the rate of trials of labor after a cesarean birth and the rate of vaginal births after a cesarean birth (VBAC) among individuals who desire a vaginal birth in a subsequent pregnancy after a cesarean birth. Historical data on inequity by race and ethnicity in cesarean birth rates have informed counseling on the mode of delivery in this population.^21^ In 2007, the Maternal-Fetal Medicine Units Network published a nomogram designed to estimate the likelihood of VBAC based on variables including maternal age, body mass index (BMI; calculated as weight in kilograms divided by height in meters squared), and race and ethnicity.^22^ The calculator estimated a lower likelihood of successful VBAC among non-Hispanic Black and Hispanic individuals.^21,23^ In recognition that inclusion of race and ethnicity in the model may further augment existing disparities,^24^ the Maternal-Fetal Medicine Units Network published an updated VBAC calculator in 2021 that eliminated race and ethnicity as a variable.^25^ We sought to examine national trends in rates of cesarean birth in the US during the period from 2012 to 2021 among nulliparous individuals and multiparous individuals with or without a prior cesarean birth. Our primary objective was to examine whether these trends differed by race and ethnicity.

## Methods

**Figure 1.**
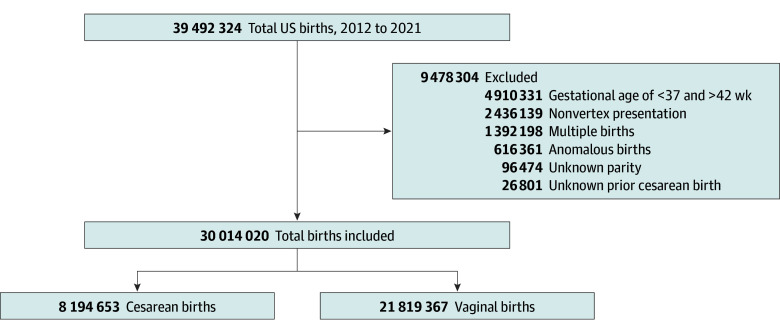
Flow Diagram Depicting Selection Criteria Birth data are from the National Vital Statistics System.

We performed a retrospective cohort study using data from linked birth registration records and infant death data collected by the National Center of Health Statistics.^26^ We included singleton, nonanomalous, full-term gestation (37 weeks and 0 days to 42 weeks and 0 days) births with vertex presentation from 2012 to 2021. We excluded individuals with unknown parity and unknown prior cesarean birth (Figure 1). This study was considered exempt from review and the requirement for informed consent by the institutional review board at Oregon Health & Science University due to the deidentified nature of the vital statistics dataset. The study followed the Strengthening the Reporting of Observational Studies in Epidemiology (STROBE) reporting guideline for cohort studies.

Our primary outcome was cesarean birth. Cesarean birth data were derived from the “method of delivery” item on the birth certificate and from information on whether the birthing individual had a previous cesarean birth. Parity was captured as nulliparous (no prior births) and multiparous (at least 1 prior birth).

Our independent variable of interest was race and ethnicity, which was self-reported by the pregnant individuals. Standards of race and ethnicity were developed by the National Center of Health Statistics working with state vital statistics organizations.^27^ Based on National Center of Health Statistics classifications, race and ethnicity were categorized into American Indian or Alaska Native, Asian or Pacific Islander, Hispanic, non-Hispanic Black, and non-Hispanic White. We used an additive modeling approach to identify race and ethnic groups, in which every self-identified category by an individual counts toward the overall measurement of these variables.^28^ In addition, we also examined parity and prior cesarean birth as effect modifiers, using 3 groups, nulliparous, multiparous with prior cesarean birth, and multiparous without prior cesarean birth. Age was categorized as younger than 20 years, 20 to 34 years, and 35 years or older. We categorized insurance as public or private, and educational level was included as a binary variable (less than college degree or a college degree and higher). Prepregnancy BMI was captured as less than 18.5 (underweight), 18.5 to 24.9 (normal weight), 25.0 to 29.9 (overweight), and 30.0 or higher (obese). We chose not to perform any multiple imputation as missing data were not substantial (<5% for any variable).

### Statistical Analysis

In the primary analysis, the proportion of cesarean deliveries was evaluated annually across the study period and examined among 3 groups: nulliparous individuals, multiparous individuals with a prior cesarean birth, and multiparous individuals without a prior cesarean birth. We graphically examined the trends in cesarean deliveries from 2012 to 2021 and used Poisson regression analyses to examine the risk of cesarean birth with an increase in birth year. Risk ratios (RRs) with 95% CIs were reported. Demographic characteristics were then compared between patients with cesarean birth and patients with vaginal birth using χ^2^ tests. Further, the proportion of cesarean deliveries along with 95% CIs were calculated among race and ethnicity groups (American Indian or Alaska Native, Asian or Pacific Islander, Hispanic, non-Hispanic Black, and non-Hispanic White) using the “proportion” command in Stata. Risk ratios (with 95% CIs) for cesarean deliveries were estimated comparing 2021 relative to 2012 in each racial and ethnic group using Poisson regression analysis. Multivariable Poisson regression analyses were then used to examine the risk of cesarean birth in 2021 vs 2012 among race and ethnic groups, after adjusting for maternal age, education, insurance, prepregnancy BMI, diabetes (preexisting and gestational), hypertension (chronic and gestational), birth weight, and gestational age.

Further, multivariable Poisson regression analyses were used to examine the risk of cesarean birth among each racial and ethnic group separately, using the same confounders. We further examined these models among nulliparous individuals and multiparous individuals with or without a prior cesarean birth; adjusted RRs (ARRs) were estimated. All regression models were clustered on state to account for any geographic differences. All proportions and RRs were calculated using Stata, version 18 (StataCorp LLC). A 2-sided *P* < .05 indicated statistical significance. Data were analyzed from March 27, 2024, to July 13, 2025.

## Results

**Figure 2.**
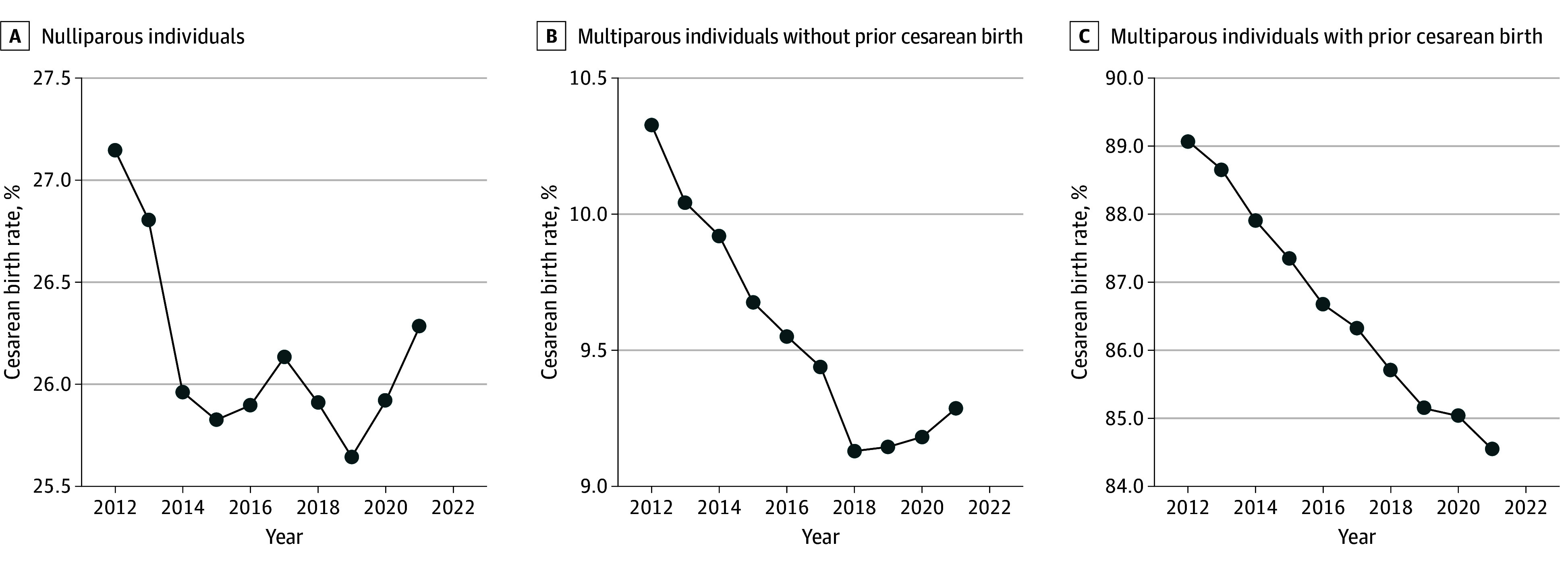
Trends in Cesarean Births by Parity and Prior Cesarean Deliveries in the US Trends in cesarean birth rates from 2012 to 2021 are shown in 3 groups.

In total, 30 014 020 births (mean [SD] maternal age, 28.6 [5.8] years) were included, of which 27.3% were cesarean births and 72.7% were vaginal births (Figure 1). In the study population, 38.9% (n = 11 695 503) were nulliparous individuals, 46.4% (n = 13 922 146) were multiparous individuals without a prior cesarean birth, and 14.7% (n = 4 396 371) were multiparous individuals with a prior cesarean birth. Cesarean birth rates decreased during the study period in all 3 groups (Figure 2). Poisson regression analyses showed that cesarean deliveries decreased in the entire cohort (RR, 0.99 [95% CI, 0.99-0.99]; *P* = .02), among multiparous individuals without a prior cesarean birth (RR, 0.99 [95% CI, 0.99-0.99]; *P* < .001), and among multiparous individuals with a prior cesarean birth (RR, 0.99 [95% CI, 0.98-0.99]; *P* < .001), whereas rates of VBAC increased (eFigure 1 in Supplement 1). Cesarean deliveries were higher among non-Hispanic Black individuals (30.9% [1 477 110 of 4 786 902]) than Asian or Pacific Islander individuals (28.0% [651 948 of 2 330 997]), and Hispanic individuals (27.6% [1 691 606 of 6 138 771]), among those with a maternal age of 35 years or older compared with 20 to 34 years (35.8% [1 785 351 of 4 988 785] vs 26.2% [6 122 056 of 23 374 323]; *P* < .001), and among individuals with a BMI of 30 or higher compared with 18.5 to 24.9 (37.8% [2 891 309 of 7 656 306] vs 21.5% [2 767 026 of 12 899 380]; *P* < .001) (Table 1).

**Table 1.** Demographic Characteristics of Participants With Nulliparous, Full-Term, Singleton, or Vertex Cesarean Births in the United States (2012-2021)

Characteristic	No./total No. (%) of participants who reported a cesarean birth (N = 8 194 653)
Maternal race and ethnicity	
American Indian and Alaska Native	112 798/450 608 (25.0)
Asian or Pacific Islander	651 948/2 330 997 (28.0)
Hispanic	1 691 606/6 138 771 (27.6)
Non-Hispanic Black	1 477 110 /4 786 902 (30.9)
Non-Hispanic White	6 119 314/23 096 430 (26.5)
Maternal age, y	
<20	287 246/1 650 912 (17.4)
20-34	6 122 056/23 374 323 (26.2)
≥35	1 785 351/4 988 785 (35.8)
Prepregnancy BMI	
<18.5	167 350/977 770 (17.1)
18.5-24.9	2 767 026/12 899 380 (21.5)
25.0-29.9	2 164 584/7 724 550 (28.0)
≥30.0	2 891 309/ 7 656 306 (37.8)
Education	
Less than college degree	4 672 636/17 452 667 (26.8)
College degree and higher	3 424 391/12 195 475 (28.1)
Insurance	
Private	4 165 875/14 818 934 (28.1)
Public	3 685 415/13 781 667 (26.7)
Pregestational diabetes	115 130/217 485 (52.9)
Gestational diabetes	672 046/1 820 089 (36.9)
Chronic hypertension	208 633/486 513 (42.9)
Gestational hypertension	597 081/1 680 518 (35.5)

Abbreviation: BMI, body mass index (calculated as weight in kilograms divided by height in meters squared).

### Cesarean Births in 2021 vs 2012 Among Racial and Ethnic Groups

Among non-Hispanic White individuals, the proportion of cesarean births decreased from 27.4% (95% CI, 27.3%-27.5%) in 2012 to 26.0% (95% CI, 25.9%-26.1%) in 2021 with an ARR of 0.87 (95% CI, 0.85-0.89). A similar finding was observed among Hispanic individuals—the cesarean birth rate decreased from 2012 (28.6% [95% CI, 28.4%-28.7%]) to 2021 (27.1% [95% CI, 26.9%-27.2%]) with an ARR of 0.88 (95% CI, 0.84-0.92)—and also among Asian or Pacific Islander individuals (ARR, 0.89 [95% CI, 0.86-0.92]) and non-Hispanic Black individuals (ARR, 0.91 [95% CI, 0.89-0.94]). There was no significant change in the rate of cesarean births among American Indian or Alaska Native individuals from 2012 to 2021 (ARR, 0.98 [95% CI, 0.90-1.07]) (Table 2).

**Table 2.** Multivariable Poisson Regression Analysis Showing Risk Ratio for Cesarean Deliveries in 2021 vs 2012

Race and ethnicity	Total No. of births	Cesarean births, No. (%) [95% CI]	Risk ratio (95% CI)	
			Unadjusted	Adjusted^a^
American Indian or Alaska Native				
2012	27 433	7046 (25.7) [25.2-26.2]	0.75 (0.74-0.75)	0.98 (0.90-1.07)
2021	46 038	11 667 (25.3) [24.9-25.7]		
Asian or Pacific Islander				
2012	186 647	52 593 (28.2) [27.9-28.4]	0.98 (0.95-1.02)	0.89 (0.86-0.92)
2021	227 020	63 194 (27.8) [27.6-28.0]		
Hispanic				
2012	560 955	160 357 (28.6) [28.4-28.7]	0.97 (0.93-1.02)	0.88 (0.84-0.92)
2021	638 996	173 018 (27.1) [26.9-27.2]		
Non-Hispanic Black				
2012	404 183	125 158 (30.9) [30.8-31.1]	1.00 (0.97-1.03)	0.91 (0.89-0.94)
2021	483 820	149 889 (30.9) [30.8-31.1]		
Non-Hispanic White				
2012	2 117 225	580 886 (27.4) [27.3-27.5]	0.95 (0.94-0.97)	0.87 (0.85-0.89)
2021	2 273 041	591 143 (26.0) [25.9-26.1]		

^a^ Adjusted for age, education, insurance, prepregnancy body mass index, diabetes (pregestational and gestational), hypertension (chronic and gestational), birth weight, and gestational age.

### Cesarean Births in Racial and Ethnic Groups

Multivariable Poisson regression analyses showed that, among the entire cohort in 2012, the risk of cesarean birth was significantly higher among non-Hispanic Black individuals (ARR, 1.12 [95% CI, 1.11-1.13]) and Asian or Pacific Islander individuals (ARR, 1.11 [95% CI, 1.07-1.16]). In 2021, the adjusted risk of cesarean birth remained high among Asian or Pacific Islander individuals (ARR, 1.10 [95% CI, 1.05-1.16]) and was even higher among non-Hispanic Black individuals (ARR, 1.17 [95% CI, 1.14-1.20]) (eTable in Supplement 1).

**Figure 3.**
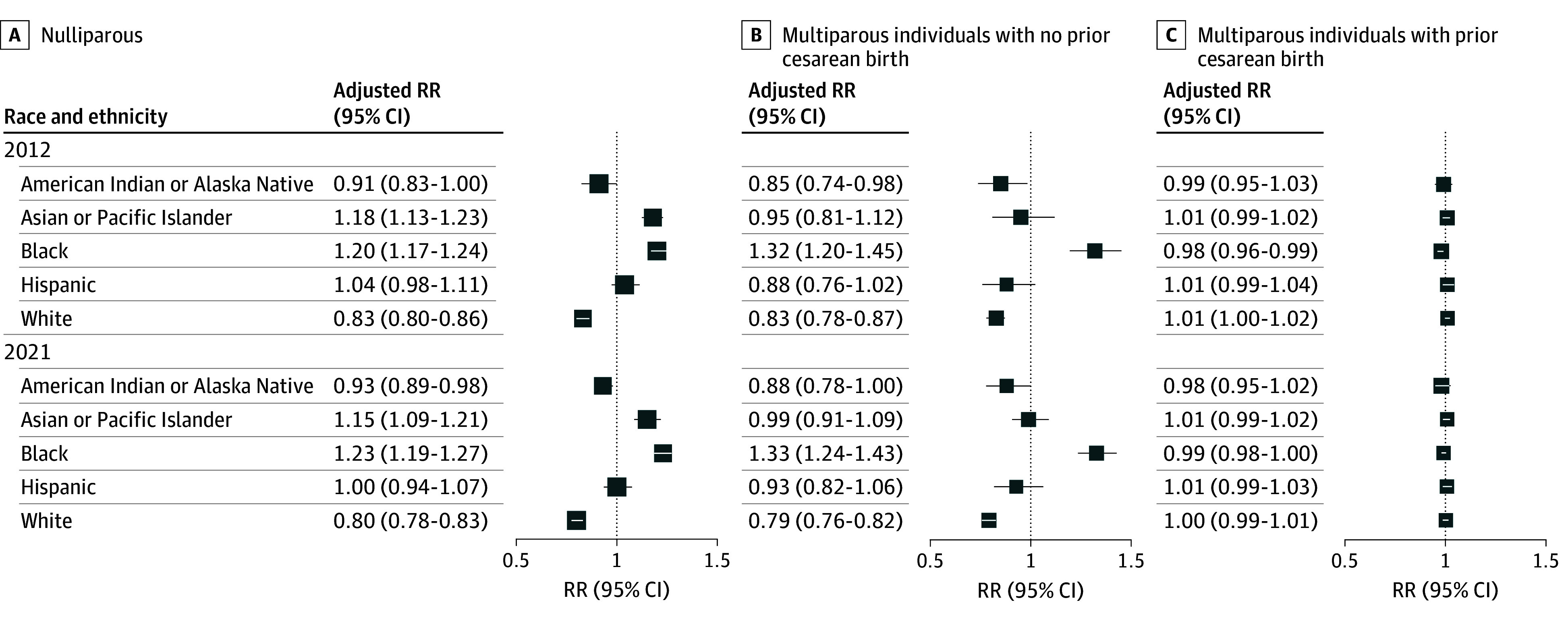
Multivariable Poisson Regression Analyses Showing Adjusted Risk Ratios for Cesarean Births in Racial and Ethnic Groups in 2012, 2021, and Overall During Study Period Adjusted risk ratios (RRs) are reported.

The finding of increased risk of primary cesarean birth among non-Hispanic Black individuals persisted when stratified by parity. A trend toward widening of this disparity was observed among non-Hispanic Black multiparous individuals between 2012 (ARR, 1.20 [95% CI, 1.17-1.24]) and 2021 (ARR, 1.23 [95% CI, 1.19-1.27]), and this disparity persisted among non-Hispanic Black multiparous individuals without a prior cesarean birth between 2012 (ARR, 1.32 [95% CI, 1.20-1.45]) and 2021 (ARR, 1.33 [95% CI, 1.24-1.43]). Among multiparous individuals with a prior cesarean birth, the adjusted risk of cesarean birth was slightly decreased among non-Hispanic Black individuals compared with individuals from other racial and ethnic groups in 2012 (ARR, 0.98 [95% CI, 0.96-0.99]); however, no difference was observed in 2021 (ARR, 0.99 [95% CI, 0.98-1.00]) (Figure 3; eFigures 2, 3, and 4 in Supplement 1).

## Discussion

Racial and ethnic disparities in the rates of cesarean birth were evident at the start and end of the study period, with increased risk among non-Hispanic Black individuals who are pregnant. Although the overall rate of cesarean births decreased, rates of primary cesarean birth among nulliparous and multiparous non-Hispanic Black individuals increased in 2012, and this disparity had further widened in 2021. An increased rate of primary cesarean deliveries was observed among Asian or Pacific Islander individuals at the start and end of the study period.

Racial and ethnic disparities in the rates of cesarean birth and attendant morbidities in specific populations within the US have been well described^29,30,31^; this study describes trends in cesarean births nationally. Our findings are consistent with previous studies demonstrating racial and ethnic inequity in cesarean birth rates, particularly among non-Hispanic Black individuals who are pregnant.^32^ Race and ethnicity are social constructs that profoundly impact the lives of individuals; observed inequities are due to structural factors such as systemic racism and implicit bias rather than individual intrinsic factors.^33^ Systemic racism^34^ drives patient-specific factors due to exposure to continuous and chronic stress, clinician-specific factors including disparities in interpretation of fetal heart rate tracings due to implicit bias,^31,35^ and population-level factors due to redlining, income and environmental inequality, and unequal access to health care.^36,37^

Non-Hispanic Black multiparous individuals with a prior cesarean birth had a slightly lower rate of a repeat cesarean birth compared with individuals from other racial and ethnic groups in 2012; this difference was no longer observed in 2021. Our study period coincides with the period during which the Maternal-Fetal Medicine Units Network VBAC calculator incorporating race and ethnicity as a variable was in use in clinical practice. In 2021 investigators published an updated calculator, removing the variables “African American” (yes or no) or “Hispanic” (yes or no), which estimated a lower likelihood of successful VBAC for non-Hispanic Black or Hispanic individuals, and adding the variable “treated chronic hypertension” (yes or no), without decreasing accuracy in the estimation of likelihood successful VBAC.^21^ Clinical decision support tools such as the VBAC calculator will inevitably be adopted variously across obstetric practices. However, the story of the evolution of the VBAC calculator is one example of structural factors that impact decision-making regarding mode of delivery.

Addressing inequities in cesarean birth rates requires a structural change within health care systems and communities. Evidence-based policy changes should be pursued.^6^ Quality improvement interventions associated with lower cesarean birth rates include continuous labor support^38,39,40,41^ and performance feedback loops for clinicians.^42^ Incorporation of practices delineated in Alliance for Innovation on Maternal Health bundles^43^ should be implemented to encourage hospitals and clinicians to reflect on clinical practice and interpersonal interactions to enact change on a local level.^6^ The use of the Robson cesarean classification system^20^ for assessment and comparison of cesarean birth rates across systems is recommended by the World Health Organization^18^ to inform research and clinical practice change; we anticipate that the implementation of this classification system^44^ will enable ongoing rigorous quality improvement efforts at a population level.

Dismantling systemic racism requires a multifaceted approach that begins with the acknowledgment of racism as the root of observed inequities and includes investment in communities that are disproportionately impacted.^34^ Addressing structural factors driving maternal health disparities requires policy change at the federal, state, and institutional level.^45^ Ongoing investment in curricular development for clinicians to address implicit bias is needed, as well as development of a clinician workforce that represents communities served across the United States.^46^ We support ongoing research examining effective interventions to prevent unindicated cesarean births and dismantle systemic racism.

### Strengths and Limitations

The strengths of this study include a large population-based sample drawn from national birth certificate data. This study also has limitations. Given the observational study design, we are unable to draw conclusions regarding causality as regards the association of structural factors with trends in racial and ethnic disparities in cesarean births and cannot exclude the potential for residual confounding. Additional limitations include the possibility of race and ethnicity misclassification on birth certificates as well as the use of singular-race inclusion criteria, which may lead to undercounting of American Indian and Alaska Native people in particular^47,48^; therefore, national trends in cesarean birth rates in this group should be interpreted with caution.

## Conclusions

In this cohort study of births in the United States from 2012 to 2021, we found that the overall rate of cesarean births among full-term singleton vertex births, irrespective of parity, plateaued and even decreased over the study period, although the rate did not reach the Healthy People 2030 target. However, this decrease in the cesarean birth rate was not observed among for non-Hispanic Black individuals, who had the highest rates of cesarean birth in 2012. The cesarean birth rate for non-Hispanic Black individuals further increased in 2021, worsening the disparity in the rate of primary cesarean births between non-Hispanic Black and non-Hispanic White individuals. A decade following the publication of care consensus guidance for the safe prevention of primary cesarean births and 4 years following the removal of race and ethnicity as a variable in the Maternal-Fetal Medicine Units Network VBAC calculator, a critical examination of racial and ethnic disparities in the national trends in cesarean birth rates is warranted. Persistent disparities in cesarean birth rates by race and ethnicity demand that quality improvement efforts to safely prevent primary cesarean births directly address the structural racism that drives these inequities.

## Notes

Supplement 1. **eTable.** Multivariable Poisson regression analyses showing association of race and ethnic categories with cesarean birth in the entire cohort (n=30,014,020) **eFigure 1.** Trends in vaginal birth after cesarean by race/ethnicity in the United States, 2012-2024 **eFigure 2.** Trends in cesarean rates in nulliparas by race/ethnicity in the United States, 2012-2021 **eFigure 3.** Trends in cesarean rates in multiparas without prior cesarean by race/ethnicity in the United States, 2012-2021 **eFigure 4.** Trends in cesarean rates in multiparas with prior cesarean by race/ethnicity in the United States, 2012-2021

Supplement 2.
Data Sharing Statement
