# Supplementary material for: Racial and Ethnic Disparities in Cesarean Birth Trends in the United States
Source: JAMA Netw Open. 2025 Nov 17;8(11):e2544078. doi: 10.1001/jamanetworkopen.2025.44078 (PMC12625682; doi:10.1001/jamanetworkopen.2025.44078)
Supplement: Supplement 1. — eTable. Multivariable Poisson regression analyses showing association of race and ethnic categories with cesarean birth in the entire cohort (n=30,014,020) eFigure 1. Trends in vaginal birth after cesarean by race/ethnicity in the United States, 2012-2024 eFigure 2. Trends in cesarean rates in nulliparas by race/ethnicity in the United States, 2012-2021 eFigure 3. Trends in cesarean rates in multiparas without prior cesarean by race/ethnicity in the United States, 2012-2021 eFigure 4. Trends in cesarean rates in multiparas with prior cesarean by race/ethnicity in the United States, 2012-2021 [file jamanetwopen-e2544078-s001.pdf]

Supplemental Online Content

Boller MJ, Garg B, Baker HA, Rodriguez MI, Marshall NE, Caughey AB. Racial and ethnic disparities in cesarean birth trends in the United States. *JAMA Netw Open*. 2025;8(11):e2544078. doi:10.1001/jamanetworkopen.2025.44078

eTable. Multivariable Poisson regression analyses showing association of race and ethnic categories with cesarean birth in the entire cohort (n=30,014,020)

eFigure 1. Trends in vaginal birth after cesarean by race/ethnicity in the United States, 2012-2024

eFigure 2. Trends in cesarean rates in nulliparas by race/ethnicity in the United States, 2012-2021

eFigure 3. Trends in cesarean rates in multiparas without prior cesarean by race/ethnicity in the United States, 2012-2021

eFigure 4. Trends in cesarean rates in multiparas with prior cesarean by race/ethnicity in the United States, 2012-2021

This supplemental material has been provided by the authors to give readers additional information about their work.

© 2025 Boller MJ et al. *JAMA Network Open.*

eTable. Multivariable Poisson regression analyses showing association of race and ethnic categories with cesarean birth in the entire cohort (n=30,014,020). Adjusted risk ratio (95% CI) reported.

	2012	2021	Overall
AIAN	0.91 (0.81-1.01)	0.92 (0.85-0.98)	0.89 (0.82-0.98)
Asian/Pacific Islander	1.11 (1.07-1.16)	1.10 (1.05-1.16)	1.11 (1.07-1.16)
Black	1.12 (1.10-1.16)	1.17 (1.14-1.20)	1.14 (1.12-1.17)
Hispanic	1.01 (0.96-1.06)	1.00 (0.94-1.06)	0.99 (0.93-1.05)
White	0.89 (0.86-0.91)	0.85 (0.83-0.87)	0.87 (0.85-0.89)

Adjusted for age, education, insurance, pre-pregnancy BMI, diabetes (pre-existing and gestational), hypertension (chronic and gestational), birthweight and gestational age.

AIAN = American Indian and Alaska Native

eFigure 1. Trends in vaginal birth after cesarean by race/ethnicity in the United States, 2012-2021

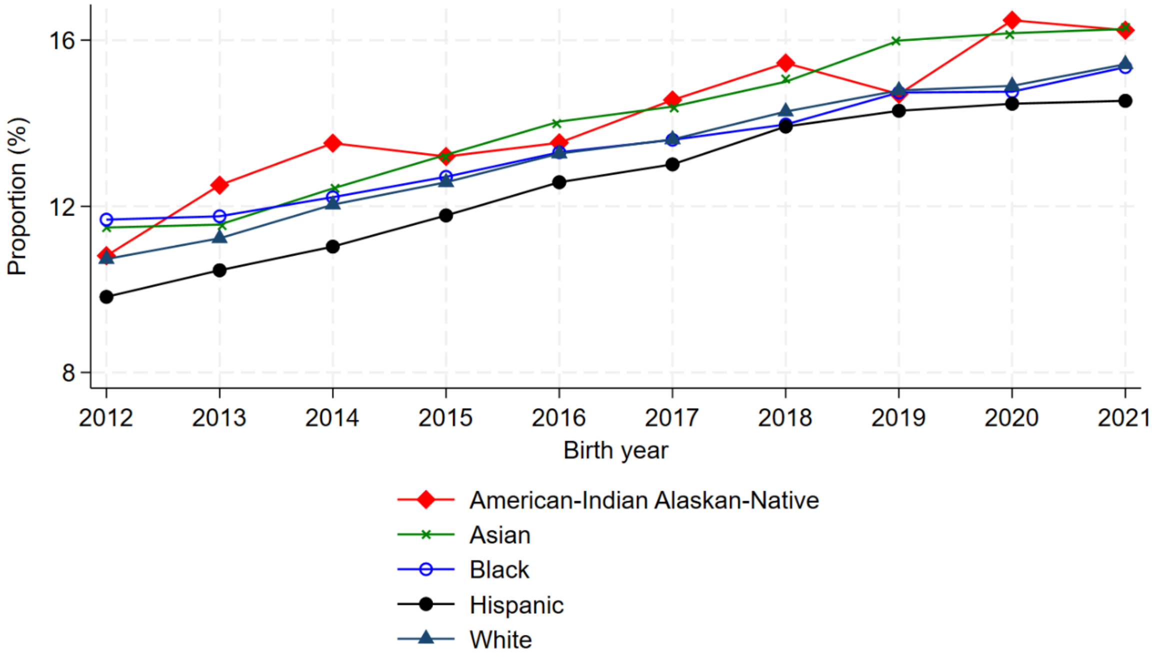

© 2025 Boller MJ et al. *JAMA Network Open.*

eFigure 2. Trends in cesarean rates in nulliparas by race/ethnicity in the United States, 2012-2021

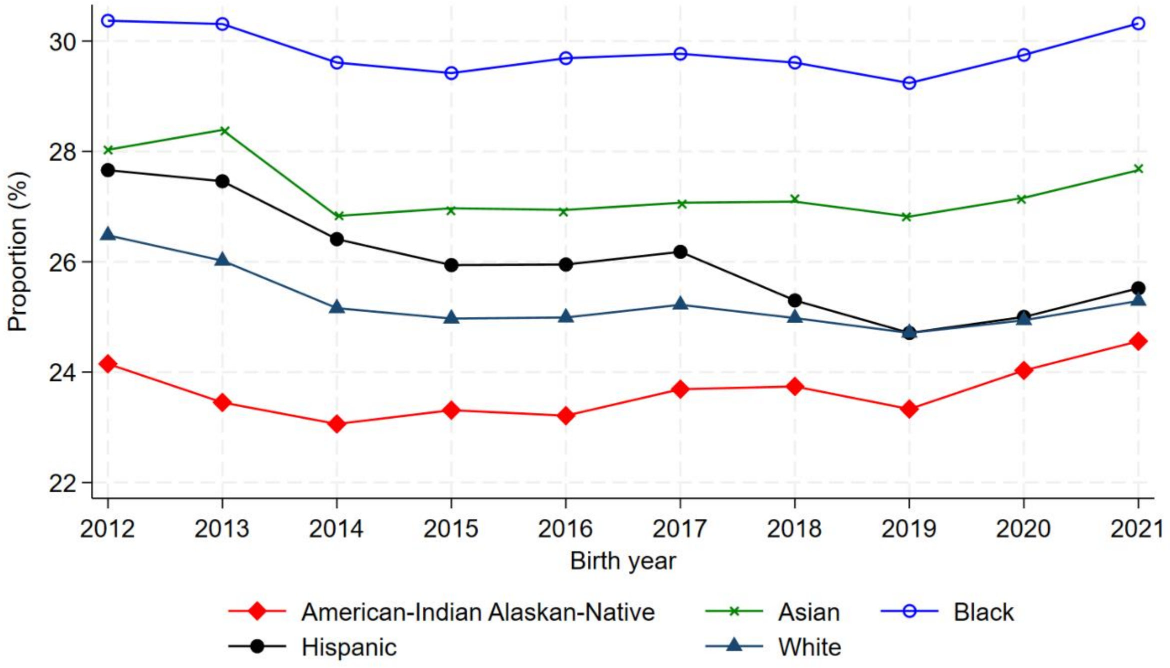

eFigure 3. Trends in cesarean rates in multiparas without prior cesarean by race/ethnicity in the United States, 2012-2021

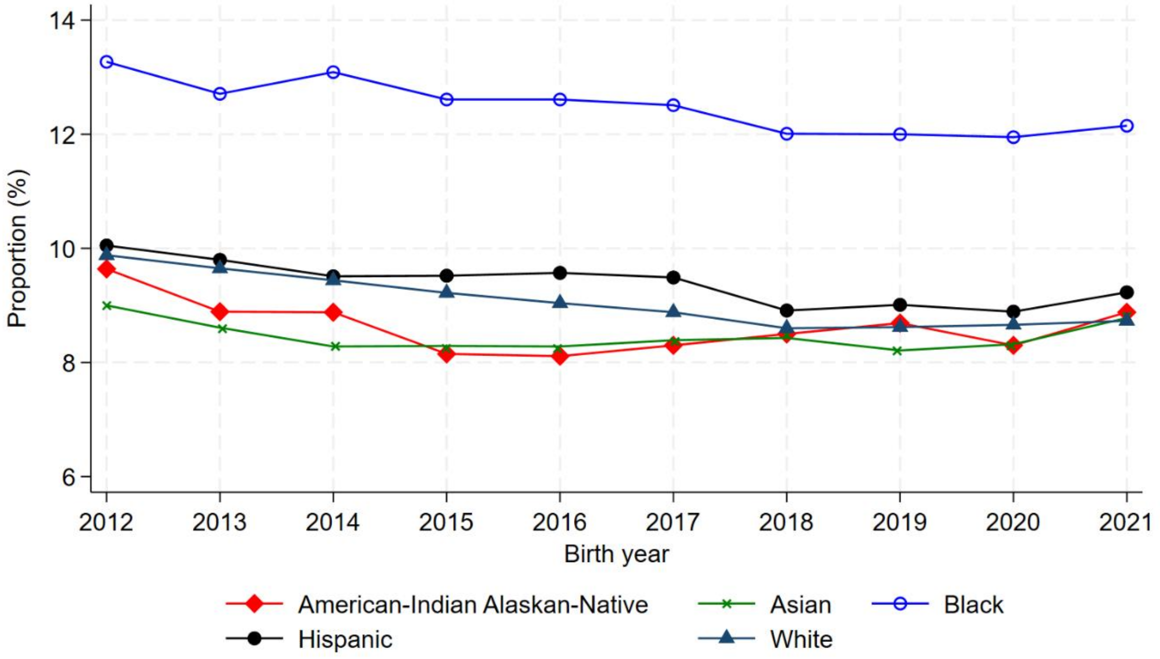

© 2025 Boller MJ et al. *JAMA Network Open.*

eFigure 4. Trends in cesarean rates in multiparas with prior cesarean by race/ethnicity in the United States, 2012-2021

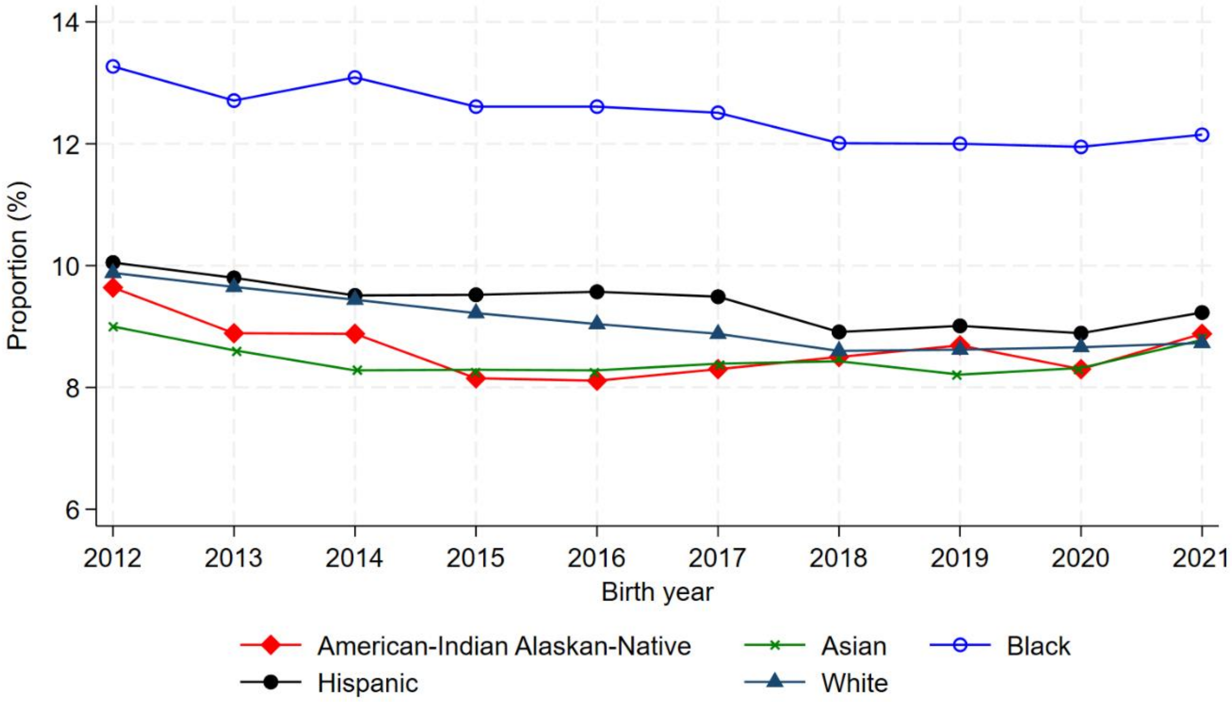

© 2025 Boller MJ et al. *JAMA Network Open.*
