# Supplementary material for: Racial and Ethnic Disparities in Cesarean Birth Trends in the United States
Source: JAMA Netw Open. 2025 Nov 17;8(11):e2544078. doi: 10.1001/jamanetworkopen.2025.44078 (PMC12625682; doi:10.1001/jamanetworkopen.2025.44078)
Supplement: Supplement 2. — Data Sharing Statement [file jamanetwopen-e2544078-s002.pdf]

Data Sharing Statement

Boller. Racial and Ethnic Disparities in Cesarean Birth Trends in the United States. *JAMA Netw Open*. Published November 17, 2025. doi:10.1001/jamanetworkopen.2025.44078

Data
Data available: Yes
Data types: Deidentified participant data
How to access data: https://www.cdc.gov/nchs/nvss/index.htm
When available: With publication

Supporting Documents
Document types: None

Additional Information
Who can access the data: anyone requesting data
Types of analyses: for any purpose
Mechanisms of data availability: National Center for Health Statistics NVSS, available to public
